# Mating-induced prolactin surge is not required for subsequent neurogenesis in male mice

**DOI:** 10.3389/fnbeh.2023.1227726

**Published:** 2023-07-06

**Authors:** Kristina O. Smiley, Hollian R. Phillipps, Chenyun Fang, Rosemary S. E. Brown, David R. Grattan

**Affiliations:** ^1^Department of Anatomy, Centre for Neuroendocrinology, School of Biomedical Sciences, University of Otago, Dunedin, New Zealand; ^2^Department of Physiology, Centre for Neuroendocrinology, School of Biomedical Sciences, University of Otago, Dunedin, New Zealand

**Keywords:** prolactin, neurogenesis, mating, paternal behavior, male mice C57BL, infanticide, BrdU, subventricular zone

## Abstract

Parenting involves major behavioral transitions that are supported by coordinated neuroendocrine and physiological changes to promote the onset of novel offspring-directed behaviors. In comparison to maternal care, however, the mechanisms underlying the transition to paternal care are less understood. Male laboratory mice are predominantly infanticidal as virgins but show paternal responses 2 weeks after mating. Interestingly, males show a mating-induced surge of prolactin, which we hypothesized may be involved in initiating this behavioral transition. During pregnancy, prolactin stimulates olfactory bulb neurogenesis, which is essential for maternal behavior. Mating induces olfactory bulb neurogenesis in males, but it is unknown whether this is driven by prolactin or is important for subsequent paternal care. New olfactory neurons are generated from cells in the subventricular zone (SVZ) and take about 2 weeks to migrate to the olfactory bulb, which may account for the delayed behavioral change in mated males. We investigated whether mating increases cell proliferation at the SVZ. Males were either mated, exposed to receptive female cues, or left alone (control) and injected with Bromodeoxyuridine (BrdU, a marker of cell division). Contrary to our hypothesis, we found that mating decreased cell proliferation in the caudal lateral portion of the SVZ. Next, we tested whether prolactin itself mediates cell proliferation in the SVZ and/or new cell survival in the olfactory bulb by administering bromocriptine (prolactin inhibitor), vehicle, or bromocriptine + prolactin prior to mating. While suppressing prolactin had no effect on cell proliferation in the SVZ, administering exogenous prolactin resulted in significantly higher BrdU-labeled cells in mated but not virgin male mice. No effects of prolactin were observed on new olfactory cell survival. Taken together, prolactin may have context-dependent effects on new cell division in the SVZ, while other unknown mechanisms may be driving the effects on new olfactory cell survival following mating.

## 1. Introduction

Parental behavior is critical for offspring survival in mammalian species. While females generally provide the majority of offspring care, for some species, including humans, males also display paternal behaviors and contribute to the raising of the young. Although substantial work has been undertaken to investigate the neurobiological mechanisms that support the onset of maternal behavior, far less is known about the mechanisms which support the onset of paternal behavior in males.

Parenting involves major behavioral transitions that are supported by a number of coordinated neuroendocrine and other physiological changes to promote the onset of novel offspring-directed behaviors (reviewed in Dulac et al., 2014). One dramatic example of this can be observed in laboratory male mice. Virgin male mice are often aggressive and infanticidal toward foreign pups, but approximately 2 weeks after mating, infanticidal behavior is suppressed and males begin to display paternal behavior for the duration of the pup-rearing period (Vom Saal, 1985; Perrigo et al., 1990, 1992). While the neural circuitry underlying infanticidal behavior (Tachikawa et al., 2013; Tsuneoka et al., 2015; Isogai et al., 2018; Sato et al., 2020; Autry et al., 2021) and paternal behavior (reviewed in Bales and Saltzman, 2016; Kohl et al., 2018) have been well-described in the mouse, there remains a fundamental gap in our understanding of what causes this delayed “switch” in behavior after mating.

The time taken to exhibit this change in behavior after mating suggests a slow, long-term plasticity is required. One hypothesis that may explain this delay is that olfactory bulb neurogenesis (the birth of new neurons) is required to make the transition from infanticidal to paternal behavior. New olfactory cells arise from neural stem cells located along the wall of the subventricular zone of the lateral ventricle (SVZ), which migrate along the rostral migration stream where they then disperse and differentiate into functional interneurons in the olfactory bulb (Lim and Alvarez-Buylla, 2016). This process takes about 2 weeks to complete—which, interestingly, mirrors the 2-week delay in the onset of mating-induced paternal behavior in male mice. Olfactory bulb neurogenesis is known to be important for parental behavior and offspring recognition in other rodent species (reviewed in Lévy et al., 2011), as well as olfactory recognition of novel odors, formation of new olfactory memories, and other olfactory-guided behavior (reviewed in Whitman and Greer, 2009; Rymer, 2020). It has recently been reported that mating induces olfactory bulb neurogenesis in male mice (Velazco-Mendoza et al., 2019). In order to gain a more complete understanding of the impacts of mating on olfactory neurogenesis, we followed up these results by testing whether mating also affects other timepoints in the mating-induced neurogenesis cascade—specifically whether mating significantly increases cell proliferation in the SVZ, which may impact future olfactory neurogenesis.

In addition to mating-induced neurogenesis, mating is also associated with a large (up to fivefold increase), transient surge in the hormone prolactin in male mice (Valente et al., 2021; Smiley et al., 2022). This surge was originally hypothesized to play a role in the male refractory period between mating bouts, however, recent work in two different strains of mice found no support for this hypothesis (Valente et al., 2021). Hence, the role of this mating-induced prolactin surge in males remains unknown. In female mice, the prolactin surges that follow mating (and occurs at the onset of pregnancy) have been shown to stimulate cell proliferation at the SVZ (the beginning stages of neurogenesis; Shingo et al., 2003), which is important for the normal onset of post-partum maternal behavior (Larsen and Grattan, 2010). Therefore, we hypothesize that the mating-induced prolactin surge in males may cause an increase in neurogenesis, which may explain the delay in paternal behavior onset. To begin testing this hypothesis, we measured whether mating-induced prolactin significantly affects (1) cell proliferation rates at the SVZ and/or cell (2) survival of new olfactory bulb cells following mating. Together, these studies will help explain how mating and mating-induced prolactin may affect neurogenesis, which will inform future studies which test for a role of neurogenesis in the transition from infanticide to paternal care in males.

## 2. Materials and methods

### 2.1. Animals

All procedures were approved by the University of Otago Animal Ethics Committee in compliance with the New Zealand Animal Welfare Act (1999). Adult C57BL/6J mice were sourced from the Taieri Resource Unit (University of Otago, Dunedin, New Zealand) from stock regularly refreshed from Jackson Laboratory (IMSR Cat# JAX:000664, RRID:IMSR JAX:000664, the Jackson Laboratory, Bar Harbor, ME, USA). Mice were housed in individually ventilated cages with shredded paper nesting material and kept in temperature-controlled rooms (22 ± 1°C) on 12:12 h reverse light/dark cycles (lights on at 20:00 h) with *ad libitum* chow and water. All animal behaviors were tested during the dark phase of the light/dark cycle under sodium lighting (McLennan and Taylor-Jeffs, 2016). Mice were 8−12 weeks of age when used.

### 2.2. Experimental design

#### 2.2.1. Characterizing the effects of mating on cell proliferation at the SVZ

Firstly, we sought to investigate whether mating caused a significant increase in cell proliferation at the SVZ relative to exposure to a female alone (without mating) and control males (no female exposure or mating). Adult virgin C57BL/6J males were initially group housed and habituated to daily handling for at least 3 weeks prior to the study in order to reduce stress associated with handling and injections during the experiment. The day before testing, males were individually housed and randomly assigned to one of three groups: mated (*n* = 8), female only control (*n* = 6), or control (*n* = 6). In the mated condition, a female stimulus was placed in the male’s home cage for 2 h. Female stimuli were reproductively experienced, ovariectomized wildtype mice which were steroid-primed by using a standard protocol of injecting estradiol (0.01 mg injection/sc, dissolved in sesame oil, vol = 0.1 ml, Sigma 815) 48 h prior and progesterone (0.05 mg injection/sc, dissolved in sesame oil, vol = 0.1 ml, Sigma P0130) 4 h before testing (Liu et al., 2020) to induce sexual behavior. All females were injected at 0900 h and mating behavior tests took place between 1,400 and 1,600 h. Only males that ejaculated during the 2-h testing period were used for brain collection. At the end of the testing period, females were removed and returned to their home cage. In the female only condition, a barrier was placed in the middle of the male’s home cage which divided the cage into 2 equal sized compartments. The barrier had small cut outs so that animals could see, hear, and smell one another, but no physical contact could take place. Males were habituated to the barrier for 1 h prior to placing a steroid-primed female in the opposite compartment from the male. Females and barriers were removed at the end of the 2-h testing period. In the control condition, males were left alone in the home cage for 2 h. In all conditions, males received an injection of BrdU (i.p., 120 mg/kg dissolved in 0.2 M phosphate buffer; Shingo et al., 2003; Mak et al., 2007; Mak and Weiss, 2010) at the end of the 2-h period, followed by 2 additional BrdU injections, 2 h apart, for a total of 3 BrdU injections across 6 h following the 2-h testing period. Animals were euthanized 30 min after the last BrdU injection and brains were later analyzed for positive BrdU immunoreactivity.

#### 2.2.2. Effects of mating-induced prolactin on cell proliferation at the SVZ

We next aimed to test whether the prolactin surge that occurs after ejaculation in male mice (Smiley et al., 2022) mediates cell proliferation at the SVZ after mating. Adult virgin C57BL/6J males were habituated to daily handling and moved to individual housing the day before testing as described above (see section “2.2.1. Characterizing the effects of mating on cell proliferation at the SVZ”). On the day of testing, males were randomly assigned to receive either bromocriptine (a D2 agonist which prevents prolactin secretion from the pituitary; 200 μg injections/sc, vol = 0.3 ml, Sigma B2134; Brown et al., 2010, *n* = 18) or vehicle injections (10% ethanol dissolved in sterile saline; *n* = 8) 1.5 h prior to adding a steroid-primed female to their cage (see Experiment description above for female stimulus protocol, “2.2.1. Characterizing the effects of mating on cell proliferation at the SVZ”). We have previously demonstrated that bromocriptine completely suppresses prolactin secretion for at least 4 h post-mating using this protocol (Smiley et al., 2022) and that this protocol markedly reduces pSTAT5 expression in the brain (a marker of prolactin receptor activation) (Brown et al., 2010; Kirk et al., 2017; Smiley et al., 2022). Only males that ejaculated during the 2-h trial were used for brain collection. A subset of the bromocriptine-treated males (*n* = 8) received an additional injection of ovine prolactin [prolactin-rescue group; 5 mg/kg injection/ip, dissolved with PBS/130 mM NaCl (pH 8), NIH NHPP; Smiley et al., 2022] immediately after ejaculation to mimic the natural mating-induced prolactin surge. At the end of the testing period all males were injected with BrdU (as described in section “2.2.1. Characterizing the effects of mating on cell proliferation at the SVZ”), and females were returned to their home cage. Males received a second injection of BrdU 2 h later, for a total of 2 BrdU injections over a 2-h period. Brains were collected 30 min after the last BrdU injection and later analyzed for BrdU immunoreactivity.

#### 2.2.3. Effects of prolactin on cell proliferation at the SVZ in virgin males

To test whether prolactin had context-dependent effects on neurogenesis, we also aimed to test whether circulating prolactin affects cell proliferation rates at the SVZ in the absence of mating. Adult virgin C57BL/6J males were habituated to daily handling and individually housed the day before testing as described in sections “2.2.1. Characterizing the effects of mating on cell proliferation at the SVZ” and “2.2.2. Effects of mating-induced prolactin on cell proliferation at the SVZ” On the day of testing, males were randomly assigned to receive an injection of ovine prolactin (*n* = 6; see section “2.2.2. Effects of mating-induced prolactin on cell proliferation at the SVZ” for details) or vehicle [PBS/130 mM NaCl (pH 8), *n* = 6]. Males then received an injection of BrdU 2 h after the prolactin/vehicle injection and a second BrdU injection 2 h later. Brains were collected 30 min following the second BrdU dose and were analyzed for BrdU immunoreactivity.

#### 2.2.4. Effects of blocking mating-induced prolactin surge on cell survival in the olfactory bulb

Finally, we investigated whether the mating-induced prolactin surge affects new cell survival in the olfactory bulb (OB). Adult virgin C57BL/6J male mice were habituated and tested in the same way as described in section “2.2.2. Effects of mating-induced prolactin on cell proliferation at the SVZ,” except only using bromocriptine (*n* = 6) or vehicle control-treated mice (*n* = 6). After the 2-h mating trial concluded, females were removed, and the males remained in their home cage alone for 2 weeks. Only males that were observed to ejaculate during the trial were used for brain collection. On day 15 post-mating, brains were collected and later analyzed for positive BrdU and NeuN immunoreactivity.

### 2.3. Mating behavior analysis

Mating behaviors were scored from videos using the program Behavioral Observation Research Interactive Software (BORIS) (Friard and Gamba, 2016), including latency to first mount and to ejaculate, the number of mounts and intromissions, and average duration of sniffing/investigating the female and mounting bouts (Smiley et al., 2022). Treatment groups were unknown to the researcher when scoring behaviors.

### 2.4. Brain collection

Mice were deeply anaesthetized with sodium pentobarbital (100 mg/kg^–1^; ip injection) before transcardial perfusion with 4% paraformaldehyde in 0.1 mol L^–1^ phosphate buffer (pH 7.4). Brains were post-fixed in the same fixative overnight before being cryoprotected in 30% sucrose solution for 2 days and stored at −80°C. Brains were cut into 3 series of 30 μm-thick sections on a freezing microtome and kept in cryoprotectant at −20°C until processing.

### 2.5. BrdU immunohistochemistry (SVZ sections)

Free floating sections were subjected to an antigen retrieval step in 2 M HCl at 37°C for 30 min. After blocking endogenous peroxidases (40% methanol and 3% H_2_O_2_ in TBS), a second blocking step was conducted in 5% normal goat serum (NGS) for 20 min at room temperature. Tissue was incubated with mouse anti-BrdU (anti BrdU Clone Bu20a, Dako, Denmark; Agilent Cat# M0744, RRID:AB_10013660) at 1:1,000 in 5% NGS for 48 h at 4°C. Sections were then treated with secondary antibody (biotinylated goat anti-mouse IgG,1:500, Vector Laboratories Cat# BA-1000, RRID:AB_2313606, Vector Laboratories, Peterborough, United Kingdom) and reacted with glucose oxidase and nickel 3,3′-Diaminobenzidine (DAB) for visualization.

### 2.6. BrdU SVZ image analysis

**FIGURE 1:**
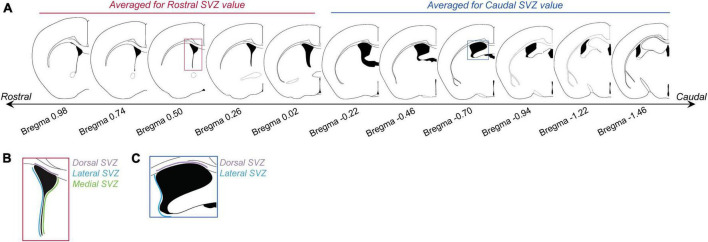
Subventricular zone (SVZ) section analysis. **(A)** Panel shows brain sections with corresponding Bregma levels that were used for analyzing BrdU cell counts in the rostral and caudal SVZ. Images based on the Mouse Brain Atlas (Franklin and Paxinos, 2013). The first five sections (Bregma levels 0.98 to –0.02) were averaged to generate “rostral SVZ” values for each animal. The last 6 sections (Bregma levels –0.22 to –1.46) were averaged to generate “caudal SVZ” values for each animal. **(B)** For each rostral SVZ section the dorsal (purple), lateral (blue), and medial (green) sections were counted and analyzed separately. **(C)** For caudal regions, only the dorsal (purple) and lateral (blue) regions were analyzed.

Images were taken on an Olympus AX70 brightfield microscope using 4 and 10X objectives. Rostral SVZ regions of interest were based on the Bregma levels most commonly analyzed in the literature (Bregma levels 0.98 to −0.02; Azim et al., 2012; Figure 1). Caudal SVZ areas, although not as commonly analyzed, also produce a number of BrdU-positive cells and thus were also analyzed (Bregma levels −0.22 to −1.46; Azim et al., 2012; Figure 1). Following the mouse atlas (Franklin and Paxinos, 2013), images were collected for the total area of the SVZ ranging from Bregma level 0.98 to −1.58, generating 22 images per animal. Sections were divided into either the rostral SVZ (Bregma levels 0.98 to −0.10) or the caudal SVZ (Bregma levels −0.22 to −1.58). For analysis, we quantified the number of BrdU-positive cells for every other image in the rostral and caudal SVZ. For each image, only one side (i.e., SVZ from the left ventricle) of the brain was counted. As it has been shown that the dorsal, ventral, and medial regions of the SVZ produce different subtypes of olfactory interneurons (reviewed in Lim and Alvarez-Buylla, 2016), we analyzed the dorsal, ventral, and medial regions of the rostral and caudal SVZ separately. Rostral and caudal SVZ were analyzed separately as generally there are more BrdU + cell in the rostral regions, compared to the caudal regions, and the only the rostral SVZ has a medial region. Positive cell labeling was automatically detected and counted using QuPath (RRID:SCR_018257) using the DAB staining algorithms tuned to custom parameters for our staining. Brains were analyzed in this way for section “2.2.1. Characterizing the effects of mating on cell proliferation at the SVZ,” “2.2.2. Effects of mating-induced prolactin on cell proliferation at the SVZ,” and “2.2.3. Effects of prolactin on cell proliferation at the SVZ in virgin males.”

### 2.7. BrdU + NeuN immunofluorescence (OB sections)

Four serial sets of sagittal brain slices (15 μm thickness) were cut through the entire brain, float-mounted onto superfrost plus slides and stored at −70°C until processing. For immunofluorescence, sections were thawed at room temperature (RT) and antigen retrieval performed using 2 M HCl at 37°C for 30 min. Endogenous peroxidases were quenched in 3% (v/v) hydrogen peroxide, 40% methanol in 0.01 M Tris Buffered Saline (TBS) and sections were then incubated in blocking solution [0.05 M TBS, 0.3% Triton X-100, 0.25% Bovine Serum Albumin (BSA), 5% normal goat serum (NGS)] for 20 min at RT. Tissue was incubated with rat anti-BrdU (Ca #AXYLL OBT0030, Bio-Rad Laboratories, Hercules, CA, USA) at 1:500 in blocking solution with 2% NGS for 16 h at 4°C, before being incubated in goat anti-rat IgG Alexa Fluor 488 (Ca #150157, Abcam, Cambridge, UK) at 1:1000 in blocking solution with no NGS for 2 h at RT. A mouse NeuN antibody (Millipore MAB377B, Merck Millipore, Burlington, MA, USA), 1:500 in blocking solution with 2% NGS for 16 h at 4°C was subsequently applied to sections to identify post-mitotic neurons. Tissue was then treated with biotinylated goat anti-mouse IgG (H + L Cross absorber, Ca#62-6540, Thermo Fisher Scientific, San Jose, CA, USA) at 1:250 in blocking solution with no NGS for 2 h at RT. Sections were immersed in avidin-biotin complex (ABC Elite kit, Vector Laboratories, Newark, CA, USA) for 90 min at RT prior to incubation with Streptavidin 647 fluorophore solution (1:400) for 2 h at 37°C. Slides were coverslipped with fluoromount aqueous medium with DAPI (Life Technologies, Carlsbad, CA, USA) to label cell nuclei.

### 2.8. BrdU + NeuN OB image analysis

Sections (10−12 per animal) were photographed using a fluorescent microscope (Nikon Ti2E Inverted Widefield with Nikon DS-Qi2 camera) under 20× objective. Images were taken within lateral coordinates 0.60 to 1.32 mm (Franklin and Paxinos, 2013) to include the glomerular and granular layer within the main and accessory olfactory bulbs (MOB and AOB). Reconstruction (5 × 5 large images) of the olfactory bulb in the XY axes was done with a motorized slide. Z-stacks (6 slices, 0.9 μm interval) were taken and images with maximum intensity were optimized from the Z-stacks using ImageJ (Rasband, W.S., ImageJ, U. S. National Institutes of Health, Bethesda, MD, USA, 1997–2018).^1^ To quantify the density of BrdU-positive and BrdU/NeuN colocalized cells in the olfactory bulb, three circular areas of 400 μm diameter were randomly placed in both the glomerular (GR) and granular layers (GL) within the MOB, and six of those with 200 μm diameter in both layers within the AOB using the software NIS-element Advanced Research 4.5 in accordance with a previously described method (Castro et al., 2022). The circular areas in the AOB were subdivided into anterior AOB (three circles per layer) and posterior AOB (another three circles per layer) based on described methods in Velazco-Mendoza et al. (2019) and Castro et al. (2022). BrdU-positive and BrdU/NeuN colocalized cells within the region of interest in the MOB and AOB were counted manually using NIS-element Advanced Research software and the researcher was unaware of treatment groups during counting. BrdU-positive and BrdU/NeuN colocalized cells were confirmed by DAPI co-expression. Two brains from each group were lost due to poor tissue quality, thus 4 males were analyzed in each treatment group. Brains were analyzed in this way for section “2.2.4. Effects of blocking mating-induced prolactin surge on cell survival in the olfactory bulb.”

### 2.9. Statistical analysis

Data were analyzed using PRISM 9 (GraphPad Prism, RRID:SCR_002798, San Diego, CA, USA). In all cases, data were checked for normality, all tests were two-tailed, and significance was accepted if *p*-values were less than 0.05.

#### 2.9.1. Characterizing the effects of mating on cell proliferation at the SVZ

The average number of BrdU-positive cell counts in the rostral and caudal SVZ were compared between mated, female-only control, and control males for the dorsal, lateral, and medial regions separately using one-way ANOVAs and Tukey’s multiple comparison *post-hoc* tests.

For males in the mated condition, mating behavior parameters (mount number, intromission number, total sniff duration, total mount duration, average sniff bout duration, average mount bout duration, latency to first mount, or latency to ejaculate) were quantified and tested for correlations with BrdU-positive cell counts in the dorsal, lateral, or medial regions of the rostral or caudal SVZ using Pearson’s *r*-test.

#### 2.9.2. Effects of mating-induced prolactin on cell proliferation at the SVZ

The average number of BrdU-positive cell counts in the rostral and caudal SVZ were compared between bromocriptine, vehicle control, and prolactin rescue control males for the dorsal, lateral, and medial regions separately using one-way ANOVAs and Tukey’s multiple comparison *post-hoc* tests.

For the mating behavior trials, the camera recording failed for some of the mating trials and therefore behavior could only be measured in 7 males in each group. Mean mating behaviors were compared between the bromocriptine-treated animals (which included animals from the bromocriptine + prolactin rescue group) and the control group using unpaired *t*-tests. Note that only these two groups were analyzed as the prolactin injection was given after mating was complete, and therefore we would not expect prolactin to affect any prior mating behavior. Latency to first mount and to ejaculate were compared between bromocriptine and control-treated animals using survival analysis and curve comparison with the Mantel-Cox Log-rank test (Swart et al., 2021; Smiley et al., 2022). Mating behavior parameters were tested for correlations with BrdU-positive cell counts in the dorsal, lateral, or medial regions of the rostral or caudal SVZ using Pearson’s *r*-test.

#### 2.9.3. Effects of prolactin on cell proliferation at the SVZ in virgin males

The average number of BrdU-positive cell counts in the rostral and caudal SVZ were compared between prolactin and vehicle-treated males for the dorsal, lateral, and medial regions separately using unpaired *t*-tests.

#### 2.9.4. Effects of blocking mating-induced prolactin surge on cell survival in the olfactory bulb

The average percentage of BrdU and NeuN positive double-labeled cells were compared between bromocriptine-treated and control males for each region of the olfactory bulb analyzed (aAOB GL, pAOB GL, aAOB GR, pAOB GR, MOB GL, and MOB GR) separately using unpaired *t*-tests.

Mean mating behaviors were compared between bromocriptine and control groups using unpaired *t*-tests. Latency to first mount and to ejaculate were compared between treatment groups using survival analysis as described in section “2.2.2. Effects of mating-induced prolactin on cell proliferation at the SVZ.” Mating behavior parameters were tested for correlations with percent Brdu + /NeuN + colocalization in each of the OB regions analyzed using Pearson’s *r*-test.

## 3. Results

### 3.1. Exposure to females and mating suppresses cell proliferation in the lateral caudal SVZ

**FIGURE 2:**
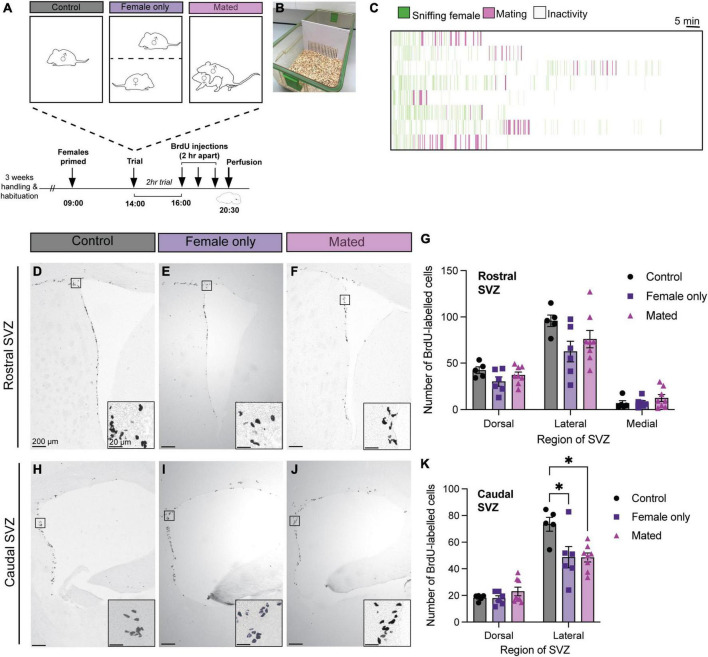
Mating and female exposure decreased cell proliferation in the caudal lateral SVZ. **(A)** Schematic of the experimental design: Adult male mice were either left in their home cage alone (control), exposed to a steroid-primed female through a barrier **(B)** (female only control), or exposed to a steroid-primed female stimulus that they mated with during a 2-h behavioral trial. All male subjects were injected with three doses of BrdU, 2 h apart, before sacrifice (30 min after the last dose of BrdU). **(C)** Ethogram of mating behaviors from the 8 males in the mating condition. Each line represents one male over the 2 h trial. **(D–F,H–J)** Representative images depict immunoreactive staining in each treatment condition (control, female only control, and mated), with insets showing higher-powered images of positive BrdU immunostaining. **(G)** There were no effects of mating or exposure to a female on cell proliferation in any of the regions of the rostral SVZ (dorsal, lateral, or medial). **(K)** There were no effects of mating in the dorsal region of the caudal SVZ. However, female-exposed and mated males showered lower BrdU staining relative to controls in the lateral caudal SVZ. *p< 0.05. Data shown in panels **(G,K)** are mean ± standard error of the mean (SEM).

There was no effect of group (mating, female-exposure only, or control) on BrdU-positive cell counts in the dorsal [*F* = 2.09_(2_, _16)_, *p* = 0.16], lateral [*F* = 2.56_(2_, _16)_, *p* = 0.11], or medial [*F* = 0.95_(2_, _16)_, *p* = 0.41] regions of the rostral SVZ (Figure 2). In the caudal SVZ, there was no effect of group in the dorsal region [*F* = 1.32_(2_, _16)_, *p* = 0.29], but there was a significant effect in the lateral region [*F* = 5.88_(2_, _16)_, *p* = 0.01] (Figure 2). *Post hoc* analysis using Tukey’s multiple comparison tests revealed that, in contrast to our predictions, both the mated (*p* = 0.02) and female-only control groups (*p* = 0.03) had lower BrdU-positive cell counts relative to the control male group (Figure 2K).

In the mated male group, none of the mating behavior parameters (mount number, intromission number, total sniff duration, total mount duration, average sniff bout duration, average mount bout duration, latency to first mount, or latency to ejaculate) were significantly correlated with BrdU-positive cell counts in the dorsal, lateral, or medial regions of the rostral or caudal SVZ (*Person’s r-test*, all *p* > 0.05).

### 3.2. Exogenous prolactin administration increases neurogenesis in mated but not virgin males

**FIGURE 3:**
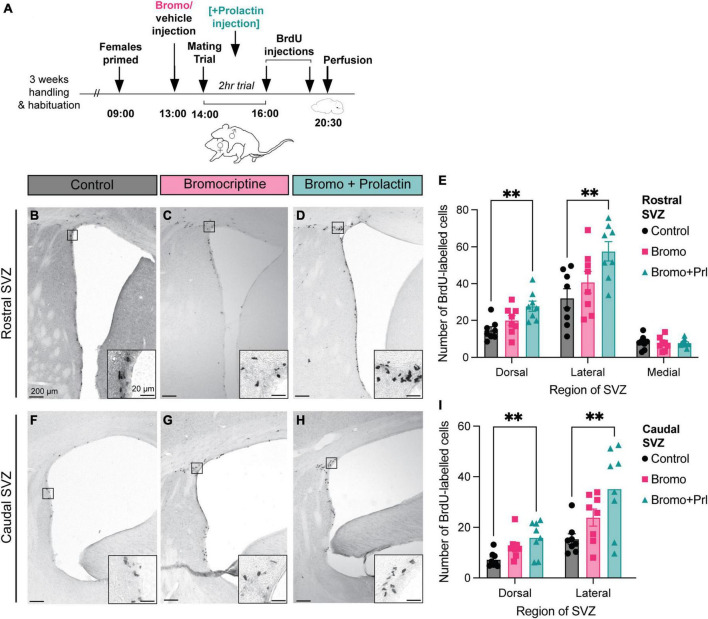
Prolactin increases cell proliferation in the SVZ in recently mated males. **(A)** Schematic of the experimental design: Adult male mice were administered an injection of bromocriptine (bromo; a prolactin inhibitor) or vehicle control 1 h before males were allowed to have a 2-h mating trial with a steroid-primed female stimulus animal. A subset of the bromocriptine-treated animals received an additional prolactin injection immediately following ejaculation to mimic the natural mating-induced prolactin surge as a prolactin rescue control group. At the end of the 2-h mating trial, all male subjects were injected with 2 doses of BrdU, 2 h apart, before sacrifice (30 min after the last dose of BrdU). **(B-D,F-H)** Representative images depict staining in each treatment group, with the insets showing higher-powered images of positive BrdU immunostaining. **(E,I)** Mated males that received a prolactin rescue injection had more BrdU positive cells in the dorsal and lateral regions of the rostral and the caudal SVZ relative to the control and bromocriptine treated males. **p < 0.01. Data shown in panels **(E,I)** are mean ± SEM.

There was no effect of treatment (bromocriptine = prolactin secretion inhibited; bromocriptine + prolactin = prolactin rescue; or vehicle control) on any of the mating behaviors measured (*One-way ANOVA*, all *p* > 0.05, Figure 3). There were no significant correlations between mating behavior durations or latencies with any of the regions in the rostral or caudal SVZ (*Pearson’s r-test*, all *p* > 0.05).

In the rostral SVZ, there was a significant effect of treatment (bromocriptine = prolactin secretion inhibited; bromocriptine + prolactin = prolactin rescue; or vehicle control) in the dorsal [*F* = 7.22_(2_, _21)_, *p* = 0.004] and lateral [*F* = 5.60_(2_, _21)_, *p* = 0.01] regions, but not the medial region [*F* = 0.09_(2_, _21)_, *p* = 0.92] (Figures 3E, I). *Post hoc* analysis revealed that there was significantly more positive BrdU immunoreactivity in the prolactin rescue group compared to the control group in both the dorsal (Tukey’s test, *p* = 0.003) and lateral (Tukey’s test, *p* = 0.01) regions of the rostral SVZ (Figure 3E). Similarly, there was an effect of treatment in the dorsal [*F* = 5.94_(2_, _21)_, *p* = 0.01] and lateral [*F* = 6.05_(2_, _21)_, *p* = 0.01] regions in the caudal SVZ, with the prolactin-rescue group having significantly higher positive BrdU immunoreactivity relative to control males in the dorsal (Tukey’s test, *p* = 0.01) and lateral (Tukey’s test, *p* = 0.01) region of the caudal SVZ (Figure 3I).

**FIGURE 4:**
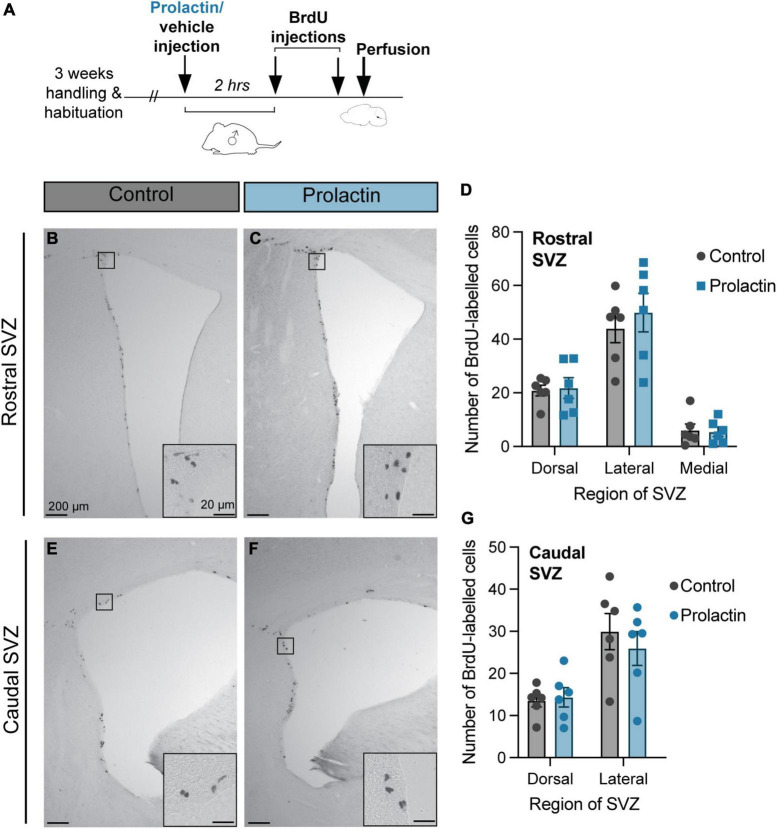
Prolactin does not increase cell proliferation in the SVZ of virgin males. **(A)** Schematic of the experimental design: Virgin males were either injected with prolactin or vehicle and left alone in their home cage. 2 h following the prolactin/vehicle injections, all male subjects were injected with 2 doses of BrdU, 2 h apart, before sacrifice (30 min after the last dose of BrdU). **(B,C,E,F)** Representative images depict staining in each treatment group, with the insets showing higher-powered images of positive BrdU immunostaining. **(D,G)** There were no effects of prolactin on cell proliferation rates in any region of the rostral or caudal SVZ. Data shown in panels **(D,G)** are mean ± SEM.

These effects of prolactin appear to be context-specific as there was no effect of treatment (prolactin or vehicle) in virgin males on positive BrdU labeling for any of the regions analyzed in the rostral SVZ: dorsal (*t* = 0.22, df = 10, *p* = 0.83), lateral (*t* = 0.67, df = 10, *p* = 0.52), or medial (*t* = 0.18, df = 10, *p* = 0.86), or the caudal SVZ: dorsal (*t* = 0.30, df = 10, *p* = 0.77), lateral (*t* = 0.68, df = 10, *p* = 0.51) (Figure 4).

### 3.3. Blocking mating-induced prolactin release had no effect on cell survival in the olfactory bulb

**FIGURE 5:**
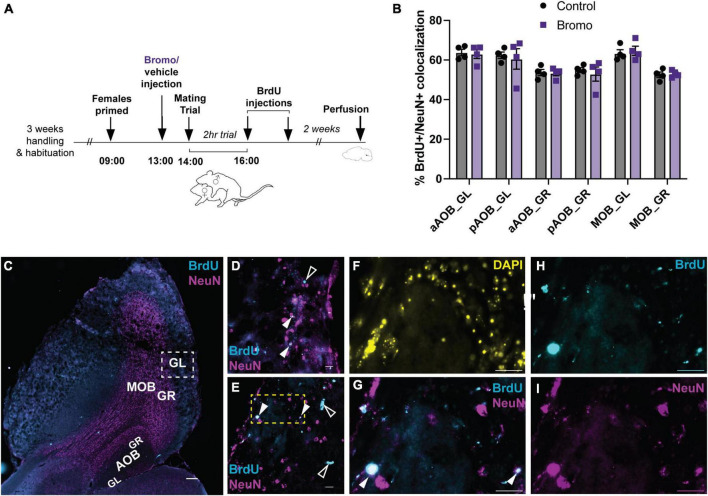
No effect of blocking prolactin on new cell survival in the olfactory bulb. **(A)** Schematic of the experimental design: Adult male mice were administered an injection of bromocriptine (bromo; a prolactin inhibitor) or vehicle control 1 h before males were allowed to have a 2-h mating trial with a steroid-primed female stimulus animal. At the end of the 2-h mating trial, all male subjects were injected with 2 doses of BrdU, 2 h apart. Brains were collected 2 weeks after the mating trial was completed. **(B)** There were no differences in the percentage of BrdU + /NeuN + immunoreactive cells between control males and bromocriptine-treated (bromo) males in any of the brain regions analyzed. aAOB, anterior accessory olfactory bulb; pAOB, posterior accessory olfactory bulb; MOB, main olfactory bulb; GL, glomerular layer; GR, granular layer. **(C)** Representative image of the olfactory bulb (sagittal plane between lateral 1.08–1.20 mm). Cyan = BrdU; Magenta = NeuN; Scale bar = 200 μm. **(D,E)** Representative 60× images from a bromo-treated **(D)** and control **(E)** mated male glomerular layer of the main olfactory bulb [area depicted by the white dashed square in **(C)**]. Filled white arrows: BrdU/NeuN colocalized cell (white); Empty while arrows: BrdU single-labeled cell (cyan). Scale bar = 20 μm. **(F-I)** Digitally magnified image from the yellow dashed region in **(E)**. **(F)**: DAPI; **(G)**: Merged Brdu (Cyan) × NeuN (Magenta); Brdu + /NeuN + colocalized cell (white; filled arrows); **(H)** BrdU; **(I)** NeuN. Scale bars = 20 μm. Data shown in panel **(B)** are mean ± SEM.

There was no effect of treatment (bromocriptine or vehicle) on the mean percentage of BrdU + /NeuN + colocalized cells for any of the olfactory bulb regions analyzed: aAOB GL (*t* = 0.27, df = 6, *p* = 0.80), pAOB GL (*t* = 0.35, df = 6, *p* = 0.74), aAOB GR (*t* = 0.14, df = 6, *p* = 0.89), pAOB GR (*t* = 0.53, df = 6, *p* = 0.62), MOB GL (*t* = 0.44, df = 6, *p* = 0.67), MOB GR (*t* = 0.32, df = 6, *p* = 0.76) (Figure 5). There was no effect of treatment on any of the mating behavior parameters measured (*t*-tests, all *p* > 0.05) or any significant correlations between mating behavior and BrdU + NeuN + colocalization (*Pearson’s r-test*, all *p* > 0.05).

## 4. Discussion

This study aimed to test whether mating and/or the mating-induced prolactin surge plays a significant role in increasing neurogenesis, via increasing SVZ cell proliferation rates and/or by increasing new cell survival in the olfactory bulb, under the hypothesis that these processes may be important for the transition from infanticide to paternal behavior. Contrary to our predictions, mating did not cause a significant increase in cell proliferation in the SVZ. Blocking the endogenous mating-induced prolactin surge did not affect SVZ cell proliferation rates, but when this treatment was rescued by a replacement injection of exogenous prolactin together with mating, we did observe a significant increase in BrdU + labeled in the SVZ. This effect appears to be context specific as we saw no differences in SVZ cell proliferation rates when exogenous prolactin was injected in the absence of mating in virgin males. Finally, blocking mating-induced prolactin release did not affect the number of new neurons (BrdU + /NeuN + labeled cells) in the olfactory bulb 2 weeks after mating.

Although mating has been reported to increase the number of newly formed neurons in the olfactory bulb of both male (Portillo et al., 2012; Unda et al., 2016; Velazco-Mendoza et al., 2019) and female (Shingo et al., 2003; Larsen and Grattan, 2010; Arzate et al., 2013; Corona et al., 2011, 2016; Alvarado-Martínez and Paredes, 2018) rodents, we did not find evidence that mating increases the rate of SVZ cell proliferation (the beginning stages of neurogenesis) in male mice. In fact, we found that cell proliferation rates in the lateral region of the caudal SVZ *decreased* following either mating or female exposure, relative to control males. In all other regions examined, there was no difference between control, female-exposed, or mated males. These findings are in contrast to male prairie voles, in which both mating and social exposure (to either a receptive female or male conspecific) *increased* the percentage of BrdU + /doublecortin + (a marker for neuron precursor cells) labeled cells in the dorsal SVZ (Castro et al., 2020). However, in that species, mating coincides with pair bond formation (which is absent in mice), and therefore, this effect may serve a different function in male voles than it would in male mice. Our findings are also in contrast to female mice, which show significantly increased SVZ cell proliferation following mating (Shingo et al., 2003; Larsen and Grattan, 2010). However, this increase in neurogenesis coincides with the initiation of pregnancy, and therefore, may not be directly comparable to mated males. For males, it may be the case that mating has a greater effect on cell survival/new cell integration in the olfactory bulb (e.g., as shown in Velazco-Mendoza et al., 2019) rather than initial cell proliferation at the SVZ.

We did find, however, that in recently mated males, exogenous prolactin administration significantly increased the number of BrdU + labeled cells in the dorsal and lateral regions of the SVZ. These results are in line with other studies which have shown that prolactin mediates cell proliferation in the SVZ. For example, in male mice, it has been shown that interactions with pups right after birth increases both SVZ cell proliferation and olfactory bulb neurogenesis, which is required for later recognition of adult offspring (Mak and Weiss, 2010). Males with a prolactin receptor (Prlr) genetic knockout or given a Prlr-neutralizing antibody failed to show this pup-induced increase in SVZ cell proliferation and were unable to recognize adult pups, suggesting this phenomenon is mediated by prolactin (Mak and Weiss, 2010). Similarly, in female mice, prolactin has been shown to mediate pregnancy-induced SVZ proliferation (Shingo et al., 2003), which influences the normal onset of maternal behavior (Larsen and Grattan, 2010), as well as mediating male pheromone-induced neurogenesis in females (Mak et al., 2007).

The effect of prolactin on increased SVZ cell proliferation appear to be context specific to mating, however, as this effect was not replicated following prolactin administration to virgin males alone. This is in contrast to other studies which have found that prolactin was able to increase cell proliferation at the SVZ in virgin males (Shingo et al., 2003; Mak and Weiss, 2010). However, these studies used infusions of prolactin via osmotic pumps over 2 days before measuring SVZ cell proliferation, whereas our study only used one acute injection of prolactin, so virgin males may need longer exposure times to high prolactin before these effects can be observed. It should be noted that unlike pregnant/lactating females, male mice would not normally experience high, sustained prolactin levels over this amount of time after mating. Prolactin is only transiently increased for ∼30 min following mating (Smiley et al., 2022), thus we were trying to more closely replicate this short increase by using acute injections rather than sustained prolactin release with pumps. It should also be noted that the prolactin injections used in this study were above the physiological level normally experienced during the mating prolactin surge and that injected levels are more similar to those observed in lactating females. So, while these results indicate that prolactin has the *ability* to increase cell proliferation at the SVZ in males, endogenous prolactin does not seem to have a role in maintaining normal levels of neurogenesis—which may explain why we did not see effects of mating of increased SVZ cell proliferation in the first study (see section “2.2.1. Characterizing the effects of mating on cell proliferation at the SVZ”). Finally, it should also be noted that BrdU is being incorporated into the DNA during the S-phase of the cell cycle in all proliferating cells in the SVZ, so we cannot determine whether there are changes in proliferation rates of certain stem cell types (e.g., neurons vs. glial cells) in SVZ, and potential differences may be there if one were to differentiate these cells with further work.

As mating has been shown to increase olfactory bulb neurogenesis in male mice (Velazco-Mendoza et al., 2019), we lastly wanted to test whether mating-induced prolactin was involved in new olfactory cell survival/integration. Contrary to our predictions, blocking prolactin secretion had no effect on new cell retention in the olfactory bulb. It should be noted that we did not have a prolactin rescue group in this experiment (see section “2.2.4. Effects of blocking mating-induced prolactin surge on cell survival in the olfactory bulb”), and therefore, it is possible that we may have seen an effect if we had administered additional prolactin at mating as we did in the previous SVZ experiment (see section “2.2.3. Effects of prolactin on cell proliferation at the SVZ in virgin males”). Previous studies have reported that olfactory bulb neurogenesis is mediated by prolactin in other (non-mating) contexts (Mak et al., 2007; Mak and Weiss, 2010). However, these studies primarily relied on the use of Prlr-/- deficient mice and not acute actions of prolactin, as we used in this study. In this context, prolactin may be more important for initial SVZ cell proliferation and not for new cell retention/survival/integration in the olfactory bulb.

Our study has primarily focused on cell proliferation in the SVZ because olfactory bulb neurogenesis has been implicated in both maternal and paternal behavior (Leuner et al., 2010; Larsen and Grattan, 2012; Rymer, 2020) and it had previously been shown that prolactin could affect both parenting behavior and SVZ/olfactory neurogenesis (Shingo et al., 2003; Larsen and Grattan, 2010; Smiley et al., 2022). However, neurogenesis certainly occurs elsewhere in the brain, such as the hippocampus and other brain regions which may also support the shift to paternal behavior (Ruscio et al., 2008; Glasper et al., 2016; Hyer et al., 2016). Future studies targeting the hippocampus could be insightful in understanding whether mating-induced neurogenesis plays a role in the parental behavioral shift in males.

Overall, while there was strong support from previous literature that mating behavior and mating-induced prolactin would increase SVZ cell proliferation and new olfactory cell survival in males, we found little evidence of this in our studies. While the endogenous rise in mating-induced prolactin had little effect on neurogenesis in the mated male, we showed that extra exogenous prolactin was able to stimulate increased cell proliferation at mating, but not in the absence of mating (i.e., in virgin males). Investigations into what other factors prolactin is interacting with at mating would be useful in order to gain a better understanding of the mechanisms driving cell proliferation in the SVZ. It is surprising that we still do not have an explanation for the role of mating-induced prolactin surge in males, and therefore additional research on this topic is warranted. Similarly, although mating-induced prolactin does not appear to affect olfactory neurogenesis outcomes, olfactory cell neurogenesis nonetheless still occurs after mating (as also previously described in Velazco-Mendoza et al., 2019). The functional consequence of this mating-induced neurogenesis has not yet been determined, however, and therefore, remains an open question in the field. Future studies could test whether blocking mating-induced neurogenesis has subsequent effects on paternal behavior, as a potential mechanism to explain the “switch” between infanticidal and paternal behavior.

## Data availability statement

The raw data supporting the conclusions of this article will be made available by the authors, without undue reservation.

## Ethics statement

The animal study was reviewed and approved by the University of Otago Animal Ethics Committee.

## Author contributions

KS: conceptualization, formal analysis, investigation, writing—original draft, visualization, project administration, and funding acquisition. HP: methodology, writing—review editing, supervision, and project administration. CF: methodology, formal analysis, investigation, writing—review editing, and visualization. RB: conceptualization, writing—review editing, and supervision. DG: conceptualization, resources, writing—review editing, and supervision. All authors contributed to the article and approved the submitted version.

## Footnotes

1
https://imagej.nih.gov/ij/

## References

[B1] Alvarado-Martínez R. Paredes R. G. (2018). Incorporation of new neurons in the olfactory bulb after paced mating in the female rat. *Behav. Brain Res.* 343 95–101. 10.1016/j.bbr.2018.02.006 29425917

[B2] Arzate D. M. Portillo W. Corona R. Paredes R. G. (2013). Repeated paced mating promotes the arrival of more newborn neurons in the main and accessory olfactory bulbs of adult female rats. *Neuroscience* 232 151–160. 10.1016/j.neuroscience.2012.12.014 23262235

[B3] Autry A. E. Wu Z. Kapoor V. Kohl J. Bambah-Mukku D. Rubinstein N. D. (2021). Urocortin-3 neurons in the mouse perifornical area promote infant-directed neglect and aggression. *eLife* 10:e64680. 10.7554/eLife.64680 34423776 PMC8452308

[B4] Azim K. Fiorelli R. Zweifel S. Hurtado-Chong A. Yoshikawa K. Slomianka L. (2012). 3-Dimensional examination of the adult mouse subventricular zone reveals lineage-specific microdomains. *PLoS One* 7:e49087. 10.1371/journal.pone.0049087 23166605 PMC3499551

[B5] Bales K. L. Saltzman W. (2016). Fathering in rodents: neurobiological substrates and consequences for offspring. *Horm. Behav.* 77 249–259. 10.1016/j.yhbeh.2015.05.021 26122293 PMC4691427

[B6] Brown R. S. E. Kokay I. C. Herbison A. E. Grattan D. R. (2010). Distribution of prolactin-responsive neurons in the mouse forebrain. *J. Comp. Neurol.* 518 92–102. 10.1002/cne.22208 19882722

[B7] Castro A. E. Domínguez-Ordoñez R. Young L. J. Camacho F. J. Ávila-González D. Paredes R. G. (2022). Pair-bonding and social experience modulate new neurons survival in adult male and female prairie voles (*Microtus ochrogaster*). *Front. Neuroanat.* 16:987229. 10.3389/fnana.2022.987229 36189119 PMC9520527

[B8] Castro A. E. Young L. J. Camacho F. J. Paredes R. G. Diaz N. F. Portillo W. (2020). Effects of mating and social exposure on cell proliferation in the adult male prairie vole (*Microtus ochrogaster*). *Neural Plast.* 2020:e8869669. 10.1155/2020/8869669 33029122 PMC7528033

[B9] Corona R. Larriva-Sahd J. Paredes R. G. (2011). Paced-mating increases the number of adult new born cells in the internal cellular (granular) layer of the accessory olfactory bulb. *PLoS One* 6:e19380. 10.1371/journal.pone.0019380 21637743 PMC3103495

[B10] Corona R. Retana-Márquez S. Portillo W. Paredes R. G. (2016). Sexual behavior increases cell proliferation in the rostral migratory stream and promotes the differentiation of the new cells into neurons in the accessory olfactory bulb of female rats. *Front. Neurosci.* 10:48. 10.3389/fnins.2016.00048 26955325 PMC4767934

[B11] Dulac C. O’Connell L. A. Wu Z. (2014). Neural control of maternal and paternal behaviors. *Science* 345 765–770. 10.1126/science.1253291 25124430 PMC4230532

[B12] Franklin K. B. J. Paxinos G. (2013). *Paxinos and Franklin’s The mouse brain in stereotaxic coordinates.* San Diego, CA: Academic Press.

[B13] Friard O. Gamba M. (2016). BORIS: a free, versatile open-source event-logging software for video/audio coding and live observations. *Methods Ecol. Evol.* 7 1325–1330. 10.1111/2041-210X.12584

[B14] Glasper E. R. Hyer M. M. Katakam J. Harper R. Ameri C. Wolz T. (2016). Fatherhood contributes to increased hippocampal spine density and anxiety regulation in California mice. *Brain Behav.* 6:e00416. 10.1002/brb3.416 27110439 PMC4834941

[B15] Hyer M. M. Hunter T. J. Katakam J. Wolz T. Glasper E. R. (2016). Neurogenesis and anxiety-like behavior in male California mice during the mate’s postpartum period. *Eur. J. Neurosci.* 43 703–709. 10.1111/ejn.13168 26750200

[B16] Isogai Y. Wu Z. Love M. I. Ahn M. H.-Y. Bambah-Mukku D. Hua V. (2018). Multisensory logic of infant-directed aggression by males. *Cell* 175 1827–1841.e17. 10.1016/j.cell.2018.11.032 30550786 PMC6558521

[B17] Kirk S. E. Xie T. Y. Steyn F. J. Grattan D. R. Bunn S. J. (2017). Restraint stress increases prolactin-mediated phosphorylation of signal transducer and activator of transcription 5 in the hypothalamus and adrenal cortex in the male mouse. *J. Neuroendocrinol.* 29 1–9. 10.1111/jne.12477 28425631

[B18] Kohl J. Babayan B. M. Rubinstein N. D. Autry A. E. Marin-Rodriguez B. Kapoor V. (2018). Functional circuit architecture underlying parental behaviour. *Nature* 556 326–331. 10.1038/s41586-018-0027-0 29643503 PMC5908752

[B19] Larsen C. M. Grattan D. R. (2010). Prolactin-induced mitogenesis in the subventricular zone of the maternal brain during early pregnancy is essential for normal postpartum behavioral responses in the mother. *Endocrinology* 151 3805–3814. 10.1210/en.2009-1385 20484459

[B20] Larsen C. M. Grattan D. R. (2012). Prolactin, neurogenesis, and maternal behaviors. *Brain. Behav. Immun.* 26 201–209. 10.1016/j.bbi.2011.07.233 21820505

[B21] Leuner B. Glasper E. R. Gould E. (2010). Parenting and plasticity. *Trends Neurosci.* 33 465–473. 10.1016/j.tins.2010.07.003 20832872 PMC3076301

[B22] Lévy F. Gheusi G. Keller M. (2011). Plasticity of the parental brain: a case for neurogenesis. *J. Neuroendocrinol.* 23 984–993. 10.1111/j.1365-2826.2011.02203.x 21824205

[B23] Lim D. A. Alvarez-Buylla A. (2016). The adult ventricular–subventricular zone (V-SVZ) and olfactory bulb (OB) neurogenesis. *Cold Spring Harb. Perspect. Biol.* 8:a018820. 10.1101/cshperspect.a018820 27048191 PMC4852803

[B24] Liu Z.-W. Jiang N. Tao X. Wang X.-P. Liu X.-M. Xiao S.-Y. (2020). Assessment of sexual behavior of male mice. *J. Vis. Exp.* 5:e60154. 10.3791/60154 32202532

[B25] Mak G. K. Weiss S. (2010). Paternal recognition of adult offspring mediated by newly generated CNS neurons. *Nat. Neurosci.* 13 753–758. 10.1038/nn.2550 20453850

[B26] Mak G. K. Enwere E. K. Gregg C. Pakarainen T. Poutanen M. Huhtaniemi I. (2007). Male pheromone-stimulated neurogenesis in the adult female brain: possible role in mating behavior. *Nat. Neurosci.* 10 1003–1011. 10.1038/nn1928 17603480

[B27] McLennan I. S. Taylor-Jeffs J. (2016). The use of sodium lamps to brightly illuminate mouse houses during their dark phases. *Lab. Anim.* 38, 384–392. 10.1258/0023677041958927 15479553

[B28] Perrigo G. Belvin L. Saal F. S. V. (1992). Time and sex in the male mouse: temporal regulation of infanticide and parental behavior. *Chronobiol. Int.* 9 421–433. 10.3109/07420529209064554 1473195

[B29] Perrigo G. Bryant W. C. vom Saal F. S. (1990). A unique neural timing system prevents male mice from harming their own offspring. *Anim. Behav.* 39 535–539. 10.1016/S0003-3472(05)80419-1

[B30] Portillo W. Unda N. Camacho F. J. Sánchez M. Corona R. Arzate D. M. (2012). Sexual activity increases the number of newborn cells in the accessory olfactory bulb of male rats. *Front. Neuroanat.* 6:25. 10.3389/fnana.2012.00025 22783170 PMC3390685

[B31] Ruscio M. G. Sweeny T. D. Hazelton J. L. Suppatkul P. Boothe E. Carter C. S. (2008). Pup exposure elicits hippocampal cell proliferation in the prairie vole. *Behav. Brain Res.* 187 9–16. 10.1016/j.bbr.2007.08.028 17913255 PMC2699755

[B32] Rymer T. L. (2020). The role of olfactory genes in the expression of rodent paternal care behavior. *Genes* 11:292. 10.3390/genes11030292 32164379 PMC7140856

[B33] Sato K. Hamasaki Y. Fukui K. Ito K. Miyamichi K. Minami M. (2020). Amygdalohippocampal area neurons that project to the preoptic area mediate infant-directed attack in male mice. *J. Neurosci.* 40 3981–3994. 10.1523/JNEUROSCI.0438-19.2020 32284340 PMC7219291

[B34] Shingo T. Gregg C. Enwere E. Fujikawa H. Hassam R. Geary C. (2003). Pregnancy-stimulated neurogenesis in the adult female forebrain mediated by prolactin. *Science* 299 117–120. 10.1126/science.1076647 12511652

[B35] Smiley K. O. Brown R. S. E. Grattan D. R. (2022). Prolactin action is necessary for parental behavior in male mice. *J. Neurosci.* 42 8308–8327. 10.1523/JNEUROSCI.0558-22.2022 36163141 PMC9653282

[B36] Swart J. M. Grattan D. R. Ladyman S. R. Brown R. S. E. (2021). Changes in maternal motivation across reproductive states in mice: a role for prolactin receptor activation on GABA neurons. *Horm. Behav.* 135:105041. 10.1016/j.yhbeh.2021.105041 34385119

[B37] Tachikawa K. S. Yoshihara Y. Kuroda K. O. (2013). Behavioral transition from attack to parenting in male mice: a crucial role of the vomeronasal system. *J. Neurosci.* 33 5120–5126. 10.1523/JNEUROSCI.2364-12.2013 23516278 PMC6705007

[B38] Tsuneoka Y. Tokita K. Yoshihara C. Amano T. Esposito G. Huang A. J. (2015). Distinct preoptic-BST nuclei dissociate paternal and infanticidal behavior in mice. *EMBO J.* 34 2652–2670. 10.15252/embj.201591942 26423604 PMC4641531

[B39] Unda N. M. Portillo W. Corona R. Paredes R. G. (2016). Sexual stimulation increases the survival of new cells in the accessory olfactory bulb of the male rat. *Front. Neurosci.* 10:65. 10.3389/fnins.2016.00065 26973447 PMC4771754

[B40] Valente S. Marques T. Lima S. Q. (2021). No evidence for prolactin’s involvement in the post-ejaculatory refractory period. *Commun. Biol.* 4:10. 10.1038/s42003-020-01570-4 33398068 PMC7782750

[B41] Velazco-Mendoza M. Camacho F. J. Paredes R. G. Portillo W. (2019). The first mating experience induces new neurons in the olfactory bulb in male mice. *Neuroscience* 396 166–174. 10.1016/j.neuroscience.2018.11.019 30471356

[B42] Vom Saal F. S. (1985). Time-contingent change in infanticide and parental behavior induced by ejaculation in male mice. *Physiol. Behav.* 34 7–15. 10.1016/0031-9384(85)90069-1 4041052

[B43] Whitman M. C. Greer C. A. (2009). Adult neurogenesis and the olfactory system. *Prog. Neurobiol.* 89 162–175. 10.1016/j.pneurobio.2009.07.003 19615423 PMC2748178

